# Surgery in Older Cancer Patients

**DOI:** 10.1007/s11912-026-01740-3

**Published:** 2026-01-24

**Authors:** Ka Yu Keith Cheung, Ruth Mary Parks, Dana Giza, Kwok-Leung Cheung

**Affiliations:** 1 Nottingham Breast Cancer Research Centre, University of Nottingham, Nottingham, England; 2 Division of Geriatric and Palliative Medicine, University of Texas Health Science Centre, McGovern Medical School, Houston, TX USA; 3 School of Medicine, University of Nottingham, Royal Derby Hospital Centre, Uttoxeter Road, Derby, DE22 3DT UK

**Keywords:** Geriatric oncology, Minimally invasive surgery, Frailty screening, Geriatric assessment, Prehabilitation, Patient optimisation

## Abstract

**Purpose of Review:**

This narrative review explores the evolving role of surgery in older adults with cancer, highlighting non-operative and minimally invasive alternatives, and the integration of geriatric principles to improve selection of treatment and postoperative outcomes.

**Recent Findings:**

Minimally invasive and non-operative treatments can provide comparable oncological outcomes to traditional surgery with lower morbidity and better functional recovery. Frailty screening tools aid in predicting postoperative outcomes, and geriatric assessment can identify vulnerabilities and assist treatment planning, prehabilitation and rehabilitation. Prehabilitation, early rehabilitation, and multidisciplinary collaboration enhance recovery and align care with patient values and outcomes that matter most.

**Summary:**

Surgical care in older cancer patients is shifting toward a model focused on preserving quality of life and personalised decision-making. Incorporating geriatric assessments and less invasive approaches can improve outcomes and reduce treatment burden. Further research is needed to integrate these strategies into standard practice.

## Introduction

As global demographics shift and life expectancy rises, the incidence of cancer in older adults continues to rise, with individuals aged 75 and over now accounting for more than one-third of new cancer diagnoses in the United Kingdom [1]. Although advancements in surgical techniques and systemic therapies have led to improved cancer outcomes across all age groups, older adults remain consistently underrepresented in clinical trials. Evidence indicates that the age gap between cancer clinical trial participants and patients newly diagnosed is both substantial and widening, raising concerns about the generalisability of trial findings to the ageing population [2].

Older patients are more likely to experience treatment-related complications and functional decline after cancer surgery [3–5]. As a result, there is growing recognition of the importance of re-evaluating the role of traditional radical surgical interventions in older adults with solid tumours, in favour of less invasive approaches. Treatment selection must not only account for traditional oncological endpoints such as survival and recurrence rates but also consider patient-centred outcomes including quality of life, functional preservation and alignment with patient preferences. Within this framework, there is growing evidence to support the integration of geriatric principles in cancer surgery decision-making. Frailty, in particular, is now widely recognised for its role as an indicator of postoperative risk, and is associated with numerous postoperative adverse outcomes, including higher mortality, longer hospital stays and increased healthcare costs in older cancer patients following surgery [6, 7].

Therefore, screening patients using frailty screening tools and geriatric assessment enables more nuanced surgical planning, supporting the selection of minimally invasive or non-operative strategies where appropriate. Additionally, growing interest in prehabilitation and postoperative rehabilitation underscores the importance of optimising physical, nutritional, and psychosocial health before and after surgery to improve recovery and maintain quality of life. Multidisciplinary collaboration between oncology and geriatric teams can help to facilitate both the delivery of geriatric assessment and the implementation of optimisation strategies.

**Fig. 1:**
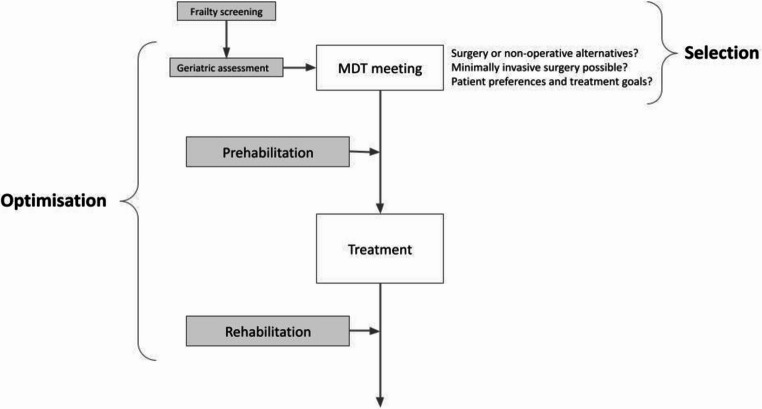
A multidisciplinary approach to surgical decision-making and perioperative care in older cancer patients. *Figure created by the authors*

This article is presented as a non-systematic, narrative review. A narrative approach was selected as the evidence surrounding surgical management of older cancer patients is highly heterogenous, with substantial variability across cancer types and treatment modalities. This narrative review aims to provide a broad overview of key themes and emerging practices in the evolving role of surgery in older adults with cancer. It explores: (1) treatment selection and the comparative outcomes of operative, minimally invasive, and non-operative strategies; (2) evaluates the role of geriatric optimisation in surgical outcomes, and (3) highlights the growing importance of multidisciplinary, patient-centred care in the older cancer population, which is summarised in Fig. 1. It is also important to acknowledge that many of the randomised controlled trials referenced in this review were not specifically designed for geriatric populations, with older frail adults frequently excluded from such studies. This limits the extrapolation of trial findings to real-world older cancer patients, and therefore should be carefully considered when interpreting the evidence presented.

## Methods

A narrative review of the literature was conducted between February and June 2025 to identify studies relevant to surgical management, non-operative alternatives, frailty assessment, and optimisation strategies in older adults with cancer. Searches were conducted on PubMed and Google Scholar. Broad combinations of terms were used, including: “older cancer patients,” “geriatric oncology,” “frailty,” “frailty screening,” “comprehensive geriatric assessment,” “minimally invasive surgery,” “non-operative management,” “prehabilitation,” “postoperative rehabilitation,” and cancer-specific terms such as “breast cancer,” “lung cancer,” “endocrine therapy,” and “axillary radiotherapy.” Reference lists of key studies and relevant review articles were manually screened to identify additional literature.

Peer-reviewed studies, systematic reviews, meta-analyses, cohort studies, clinical trials, and guideline documents were prioritised, with preference given to literature published in English within the last 5 years. As this was a non-systematic review, no formal inclusion or exclusion criteria were applied.

## Selection for Treatment

### Frailty and Treatment Selection

Frailty is a common syndrome in older adults, and is defined as a “multidimensional and dynamic condition characterised by declines in reserve and function across multiple physiological systems, such that the ability to cope with every day or acute stressors becomes compromised” [8]. Frailty has been demonstrated to be a significant predictor of postoperative complications and mortality for various cancers [6]. Measuring the degree of frailty for individual patients can therefore provide important information on treatment tolerance, helping guide selection of treatment modalities.

#### Frailty Screening Tools in Treatment Selection

Frailty screening tools are brief assessments designed for use in clinical settings to identify patients who may benefit from a more in-depth geriatric assessment. Currently, common frailty screening tools frequently used in practice include the Clinical Frailty Score (CFS), the Geriatric 8 (G8) screening tool and the Vulnerable Elders Survey (VES-13). Higher frailty screening scores are significantly associated with increased postoperative complications, longer postoperative hospital stays, readmission, morbidity and mortality, and have the ability to predict mortality as well as poor survival [9–11]. Screening tools are relatively easy and quick to perform, with the G8 having a proven diagnostic accuracy in large independent cohorts for older cancer patients [12]. Frailty screening tools are recommended by the International Society of Geriatric Oncology (SIOG) and American Society of Clinical Oncology (ASCO) guidelines [10] for use in older adults with cancer.

#### Geriatric Assessment in Treatment Selection

The Comprehensive Geriatric Assessment (CGA) is defined as “a technique for multidimensional diagnosis of frail elderly people with the purpose of planning and/or delivering medical, psychosocial, and rehabilitative care” [13]. Geriatric assessments involve the evaluation of key areas, including functional status, physical performance and falls, comorbid medical conditions, depression, social activity/support, nutritional status and cognition, which can allow clinicians to identify vulnerabilities in older individuals not found in typical oncology assessments [14]. Individualised treatment plans can be developed and can facilitate shared decision-making between clinicians and patients, aligning surgical goals with patient priorities [15].

Various components of the CGA are linked to postoperative complications in older cancer patients, including impairments in instrumental activities of daily living (IADLs), depression, cognitive decline and lower frailty composite scores. Additionally, other domains such as nutrition and polypharmacy have been demonstrated to be effective predictors of longer hospital stays [16]. Therefore, CGA can serve as a valuable tool in preoperative risk stratification and aid in treatment planning.

### The Role of Surgery in Older Cancer Patients (Table 1*)*

Surgery is the mainstay of treatment for solid malignancies in adults. When selecting treatment options for cancer in older patients, there are several possible alternatives to radical surgery to consider including: non-operative treatment, minimally invasive treatment, or no treatment. In order to select the appropriate treatment for each patient, a wide range of factors must be considered, including cancer-specific outcomes as well as patient-specific outcomes. Survival rates, recurrence rates and disease-free survival (DFS) are traditional endpoints crucial in assessing the efficacy of treatment, particularly regarding disease control and the potential for remission. However, patient-centred outcomes, including quality of life, preservation of functional status and maintenance of independence are important postoperative outcomes to consider, especially in the geriatric population. Therefore, evaluating whether comparable oncological and patient-centred outcomes can be achieved through less invasive approaches is essential, and, if surgery is deemed necessary, whether a less radical approach could be utilised.

**Table 1:** Summary of key surgical considerations for older cancer patients

Operative strategies	Minimally invasive surgery	Non-operative strategies
Frailty screening and geriatric assessment can identify high-risk older adults and allow guided patient optimisation strategies, including prehabilitation and rehabilitation [9–12, 17–20].	Consider laparoscopic and robot-assisted approaches to reduce surgical trauma and length of hospital stay [21–25].	Consider for frail/comorbid patients who may not tolerate surgery well [26–30].
Weigh the benefits and risks of upfront surgery vs. neoadjuvant systemic therapy on a case-by-case basis, accounting for patient functional status and ability to tolerate treatment [31].	Minimally invasive surgery is associated with reduced pain and faster recovery, facilitating earlier mobilisation and functional recovery [25, 32, 33].	Selection of treatment should reflect patient goals, which can vary across age and cancer type [34–36].
Multidisciplinary team working involving geriatrics improves individualised care and postoperative outcomes [37, 38].	Lowers the risk of postoperative complications older adults are more susceptible to, including delirium and venous thromboembolism [39–41].	A number of non-operative strategies have demonstrated oncological equivalence to traditional surgery while providing benefits to quality of life [26–30, 42].

#### Non-operative Therapies Versus Surgical Treatment

Non-operative treatment options are important to consider in the treatment of older cancer patients, particularly those with comorbidities or functional limitations, which may cause them to be less suitable candidates for surgery. Key examples include radiation therapy and endocrine therapy, which provide viable non-operative alternatives to surgery. An overview is given in Table 2. 

The role of radiation therapy has also increased over the years, one such example being in breast cancer in older adults. Traditionally, axillary lymph node dissection (ALND) has been performed in the setting of breast cancer when a patient is found to have nodal involvement. ALND can lead to chronic pain, numbness, lymphoedema, infection and inflammation [43]. Axillary radiotherapy (ART) has been established as an effective alternative to ALND in patients with clinically node-negative, sentinel lymph node-positive breast cancer [44]. Ten-year follow-up data from the AMAROS (After Mapping of the Axilla: Radiotherapy or Surgery) clinical trial demonstrated comparable oncological outcomes between ART and ALND, with low axillary recurrence rates (1.82% for ART vs. 0.93% for ALND), and no significant differences in overall survival (OS) (81.4% vs. 84.6%) or disease-free survival (70.1% vs. 75.0%). Importantly, ART was associated with significantly lower rates of lymphoedema (11.9% vs. 24.5%) [26, 27].

Another example of a non-operative approach is stereotactic body radiation therapy (SBRT) to treat lung cancer. Currently, surgery is the standard treatment for lung cancer, particularly non-small cell lung cancer (NSCLC). SBRT involves the administration of high ablative radiation doses to tumours while minimising exposure to surrounding healthy tissues. Compared to surgery for early-stage NSCLC, SBRT is associated with lower acute toxicity and morbidity, with grade ≥ 3 toxicity within 6–12 months reported in only 10% of cases compared to 44% for surgery [45]. Furthermore, SBRT is also associated with favourable quality of life outcomes, with no significant decrease in pulmonary function or patient-reported quality of life reported after treatment [29]. However, when survival outcomes are considered, numerous studies continue to demonstrate favourable OS for surgery in operable patients with early-stage NSCLC. For example, Miki et al. [46] reported 3-year OS of 90.1% with surgery vs. 70.5% with SBRT, while Park et al. [47] reported 5-year OS of 72.4% vs. 40% for patients ≥ 75. However, it is important to acknowledge that due to high heterogeneity within the patient population, it is possible that patients selected for operation may have a higher physiological reserve compared to patients undergoing SBRT, which may influence results. Currently, surgery remains a mainstay of management for operable lung cancer, while SBRT may be an option in older patients who are at higher risk of postoperative complications, or those ineligible for surgery [26].

Another non-operative approach in breast cancer is primary endocrine therapy (PET), which involves targeting hormone-receptor-positive breast cancers by interfering with endocrine signalling or by blocking hormone synthesis [48]. The pivotal 2006 Cochrane review by Hind et al. [42] compared PET alone to primary surgical therapy (PST) (use of surgery alone or surgery with adjuvant endocrine therapy) in women ≥ 70 with operable primary breast cancer. Notably, no statistically significant difference in overall survival between PET and PST was seen, although PET was significantly inferior to PST for progression-free survival after 5 years. A recent 2023 meta-analysis by Chan et al. [30] re-evaluated PET vs. PST in older women (≥ 65) with breast cancer and observed that PET was associated with a worse OS. However, when restricting analysis to randomised controlled trials (RCTs) and prospective studies, the OS between PET and PST was comparable, suggesting the difference in survival could be due to confounding by age and co-morbidities. PET could represent a viable non-operative alternative in carefully selected older patients. Table 2. 

**Table 2:** Summary table of quantitative effects for non-operative approaches vs. surgery

	Outcome measure	Quantitative result(s)	Risk ratio (RR)	Odds ratio (OR)	Number needed to treat/number needed to harm (NNT/NNH)
ART vs. ALND in sentinel node–positive breast cancer (AMAROS, 10-year)	Overall survival	ART 81.4% vs. ALND 84.6%^1^	-	-	-
	Disease free survival	ART 70.1% vs. ALND 75.0%^1^	-	-	-
	Lymphoedema (updated 5-year follow-up)	ART 11.9% vs. ALND 24.5%^1^	0.49	0.42	8
	Axillary recurrence	ART 1.82% vs. ALND 0.93%^1^	1.956	1.976	112
SBRT vs. surgery for early-stage NSCLC	3-year overall survival	Surgery 90.1% vs. SBRT 70.5%^2^	-	-	-
	5-year overall survival in patients ≥ 75 yrs	Surgery 65.9% vs. SBRT 40.3%^3^	-	-	-
	≥Grade 3 toxicity (6–12 months)	SBRT 10% vs. surgery 44%^4^	0.23	0.14	3
	Pulmonary function after treatment	No significant decline with SBRT^5^	-	-	-
	Quality of life after treatment	Stable quality of life with SBRT^6^	-	-	-
Primary Endocrine Therapy (PET) vs. Primary Surgical Therapy (PST)	Overall survival (RCTs and prospective only)	Comparable OS (RCTs hazard ratio = 1.12, prospective studies hazard ratio = 2.15^7^)			
	Overall survival (all studies)	PET inferior to PST (median 5-year OS PET 59.5% vs. PST 67.4%, median 10-year OS PET 24.7% vs. PST 37.7%^7^)			

^1^ Data from Bartels et al. (2023) [27]

^2^ Data from Miki et al. (2023) [46]

^3^ Data from Park et al. (2021) [47]

^4^ Data from Chang et al. (2015) [45]

^5^ Data from Videtic et al. (2013) [29]

^6^ Data from Chen et al. (2016) [49]

^7^ Data from Chan et al. (2023) [30]

#### Upfront Surgery Versus Neoadjuvant Systemic Therapy

Systemic therapies are a cornerstone of modern cancer management in younger patients. Neoadjuvant systemic therapy involves the preoperative administration of treatment such as chemotherapy or radiotherapy with the aim of downstaging tumours to improve surgical outcomes. Currently, neoadjuvant chemotherapy (NAC) has demonstrated superior survival outcomes compared to upfront surgery in a range of cancers [50, 51].

However, the use of NAC in older adults presents significant challenges due to the prevalence of comorbidities and increased susceptibility to treatment-related toxicity within this population. A cohort study by Keywani et al. [31] evaluated the role of NAC in patients aged ≥ 75 years undergoing gastrectomy for gastric adenocarcinoma. No significant difference in OS was seen between patients who received NAC compared with those undergoing upfront surgery. Notably, the study reported that patients ≥ 75 were significantly more likely to not proceed with surgery following chemotherapy compared to patients ≤ 75 (15.6% vs. 8.9%), likely due to higher rates of functional decline within the older population.

Therefore, in the treatment of older cancer patients who are borderline candidates for chemotherapy but are fit enough to undergo surgery, upfront surgery followed by adjuvant therapy may be more appropriate. Upfront surgery allows local control to be guaranteed as well as accurate pathological staging of the cancer, which could then help guide selection for adjuvant systemic therapy, reducing the risk of toxicity and morbidity.

#### Minimally Invasive Surgery Versus Traditional Open Surgery

With modern advancements in technology, novel surgical techniques such as endoscopic, laparoscopic and robotic-assisted approaches have become more prevalent, and demonstrate a less radical approach, which can help reduce postoperative complications. Minimally invasive surgery involves the use of small incisions and specialised instruments to perform operations, reducing trauma when compared to traditional open surgery.

Laparoscopic procedures are an example of minimally invasive surgery and are currently well-established in the treatment of various cancers. In colon cancer, the safety and efficacy of laparoscopic surgery has been demonstrated in a number of randomised controlled trials, including the COLOR (Colon Cancer Laparoscopic or Open Resection) [23], CLASICC (Conventional versus Laparoscopic-assisted Surgery in Colorectal Cancer) [21], COSTSG (Clinical Outcomes of Surgical Therapy Study Group) [24] and Barcelona clinical trials [22]. Collectively, these studies reported comparable outcomes between laparoscopic and open surgery in regard to resection margins, local recurrence, OS and DFS. Importantly, laparoscopic surgery was associated with a number of favourable perioperative outcomes, including a reduced use of narcotics and analgesics, shorter hospital stays and an earlier return of gastrointestinal function post-surgery [25]. However, it is important to note that none of these studies were targeted at an older cancer population.

Robotic surgery is another area of significant advancement in minimally invasive surgical approaches, particularly in prostate cancer. Open radical prostatectomy (RP) is the traditional approach to prostate surgery, and involves an 8–10 cm incision [52], while robotic-assisted laparoscopic radical prostatectomy (RARP) involves six 1–3 cm incisions [53]. The LAPRO (LAParoscopic Prostatectomy Robot Open) prospective non-randomised trial compared RARP and open retropubic radical prostatectomy (RRP) for prostate cancer in patients < 75, identified several short-term benefits of RARP compared to RRP. These benefits include reduced perioperative bleeding (185 vs. 683 mL), shorter hospital stays (3.3 vs. 4.1 days) and lower rates of reoperation during initial hospital stay for RARP compared to RRP (0.7% vs. 1.6%), although operating times were longer (175 vs. 102 min) [32]. Additionally, eight-year follow-up data demonstrated the long-term oncological safety of RARP, reporting significantly lower prostate cancer-specific mortality (PCSM) for RARP compared to RRP (1.5% vs. 2.8%). Erectile dysfunction was also significantly lower for RARP compared to RRP (66% vs. 70%) [33]. In appropriately selected older patients, minimally invasive approaches like RARP could provide similar advantages in terms of postoperative recovery and complication reduction.

The increasingly widespread use of minimally invasive approaches, including laparoscopic and robotic-assisted surgery, has expanded the number of surgical options available. A wide range of these techniques have demonstrated oncological equivalence to traditional open surgery while offering improvements in patient outcomes. This is particularly important in older adults, who are at significantly higher risk of complications such as venous thromboembolism and postoperative delirium [39, 40], largely due to the impact of radical surgery on already diminished physiological reserves [41]. Invasive procedures can lead to increased postoperative pain, hindering early mobilisation after surgery and therefore increasing the risk of complications such as deep-vein thrombosis and pulmonary embolism [19, 54]. Moreover, older patients are more susceptible to cognitive complications after surgery, with frailty severity and operative stress linked to an increased risk of postoperative delirium [55, 56]. While minimally invasive surgery offers benefits across all age groups, it is especially advantageous in older patients. By reducing surgical trauma and postoperative pain, minimally invasive surgery facilitates earlier mobilisation, shortens hospital stays, and ultimately lowers the risk of postoperative complications.

#### Non-operative Strategies

It is important to note that the decision by older adults to decline treatment is not uncommon, and involves a wide range of clinical, sociodemographic and psychosocial factors. Due to the heterogeneity of the older cancer population and limited research, determining these factors remains challenging. A 2015 systematic review by Puts et al. [34] aimed to identify factors influencing older patients’ decisions to accept or decline cancer treatment in patients with breast and prostate cancer. Key reasons for refusal included fear of side effects, anticipated treatment-related discomfort, and logistical barriers such as transportation, while factors important for accepting treatment were convenience, treatment success rate, understanding the need for treatment and physician recommendation.

### Gaps in the Evidence Base for the Treatment of Older Adults

Although the prevalence of age restrictions in clinical trials appears to be decreasing in recent years [57], age-based disparities among cancer trial participants continue to rise [2]. Restrictions dependent on organ function, co-morbidities and previous cancer limit trial participation for older adults [57], resulting in consistent underrepresentation. In particular, those > 80 and severely frail patients are commonly excluded from clinical trials, leaving limited evidence to guide their treatment. Consequently, treatment recommendations for the oldest and frailest patients vary across clinicians, and a number of complex clinical scenarios unique to older adults remains inadequately addressed in existing guidelines.

One particular challenge is the management of older cancer patients with dementia. Older cancer patients with dementia are more likely to be excluded from clinical trials due to concerns about capacity and informed consent, and therefore limit the ability to form guidelines for the management of this population [58]. Furthermore, communication difficulties also further complicate the ability for clinicians and caregivers to understand patient preferences. A study by Harrison-Dening et al. reported that while caregivers and patients with dementia had similar agreement for treatment preferences in the present moment, there was low agreement for hypothetical situations of advanced cancer, and a high level of uncertainty regarding end-of-life treatment preferences [59]. Regarding treatment decision-making, a 2021 mixed-studies review by Caba et al. [60] reported that older cancer patients with dementia experience a higher mortality compared to those without, and were less likely to receive curative treatment, including surgery, chemotherapy, and radiation therapy. A lack of evidence surrounding how different treatment options affect survival in cancer patients with dementia was also reported, and therefore has led to inconsistent approaches in treatment decision-making [61]. Future research should aim to compare outcomes between different treatment types for cancer patients with dementia, and further investigate patient preferences.

## Optimisation

If surgery is deemed an appropriate treatment, optimising the patient’s condition is essential, especially in frail patients. As mentioned previously, numerous studies have demonstrated that frailty is a significant predictor of postoperative complications and mortality [6], and therefore identifying frailty and improving physiological status is a key consideration when optimising older cancer patients before and after surgery.

### Geriatric Assessment in Patient Optimisation

Geriatric assessment also plays a crucial role in patient optimisation. A 2023 study by Montroni et al. [17] on 625 patients ≥ 70 identified key predictors of quality of life and functional recovery following colorectal cancer surgery. Factors such as a high frailty score on the Flemish version of the Triage Risk Screening test (fTRST ≥ 2), poor Eastern Collaborative Oncology Group performance status (ECOG PS ≥ 2), severe postoperative complications and a high Charlson Age Comorbidity Index (CACI ≥ 7) were strongly associated with poor functional recovery. An fTRST score ≥ 2, ECOG PS ≥ 2 and postoperative complications were also linked to reduced quality of life. Therefore, incorporating CGA can help plan appropriate timely interventions to improve outcomes. By identifying vulnerabilities associated with poor functional recovery, prehabilitation and rehabilitation strategies can be targeted at improving particular domains, supporting earlier return to baseline function and quality of life.

While CGA is a valuable tool in evaluating older adults, particularly those with cancer, its implementation presents certain challenges. In particular, a full CGA requires approximately 1–2 h to complete [62] and therefore, due to limited time, may be difficult to include in clinical practice. The CGA is also resource-intensive, requiring interdisciplinary teamwork between healthcare professionals to conduct [63]. Additionally, given the variability in how CGA are conducted, assessing the efficacy of CGA is challenging, with no universally accepted standardised approach. CGA is also potentially associated with increased healthcare cost, although the evidence is of low certainty and therefore the cost-effectiveness of CGA is uncertain [64]. However, the cost of implementing GA is comparatively lower than the expenses of imaging [65], and by managing toxicity or addressing postoperative complications it can potentially reduce hospital stay length, further lowering healthcare costs. A 2023 UK model-based economic evaluation by McKenzie et al. [66] aimed to assess the cost-effectiveness of geriatric assessment prior to cancer treatment in a number of implementation configurations. Their results showed that the implementation of geriatric assessment is associated with additional costs of between £390 and £576. In particular, the preoperative model, where GA was only delivered prior to surgery, was found to be consistently cost-effective but only if the assumed benefit in reducing postoperative complications was preserved, and only GA delivered prior to chemotherapy cost-effective even when there is no effect on reducing toxicity. Furthermore, overall uncertainty was high, with a 44% probability that GA was not cost effective (incremental net health benefit < 0), with a substantial cost of uncertainty of 2.81–2.91 quality-adjusted life years. As a result, current evidence indicates GA may only be cost-effective within certain settings, and due to differences in implementation between centres and heterogeneity, uncertainty remains high. Therefore, McKenzie et al. suggest that the implementation of GA into oncology may be better guided by local evaluation rather than generalised research. Moreover, the study was performed in the UK, and so differences in healthcare systems between countries may influence implementation of GA, affecting cost-effectiveness.

Treatment burden is also important to consider when assessing the advantages and disadvantages of CGA. Defined as the impact on a patient’s functioning and well-being caused by the demands of managing their healthcare [67], CGA has the potential to reduce treatment burden through reducing overtreatment and unnecessary appointments. A 2006 study by Keating et al. [68] reported that older women who underwent primary surgical therapy had a median number of 11, 8 and 3 face-to-face office visits during surveillance years 1, 2 and 3, respectively. As a result of frequent contact with the healthcare system, these patients may have a higher exposure to unnecessary tests in addition to their routine testing, mammograms, primary care appointments and therapy monitoring appointments, increasing the time, effort and financial burden on the patient.

### Prehabilitation and Rehabilitation

It is important to recognise that even minimally invasive procedures can lead to meaningful declines in functional status for older patients. Although breast cancer surgery is often perceived as being less intense when compared to other types of surgery due to its non-visceral nature, the extent of surgery has been shown to significantly affect postoperative outcomes. A 2021 systematic review by Harrison et al. [69] on the impact of breast cancer surgery on the functional status of older women reported that physical activity worsened with increased complexity of surgery. Decreases in the ability to perform IADLs and quality of life in older patients after breast cancer surgery have also been observed, further highlighting the importance of both preoperative and postoperative patient optimisation [4].

#### Prehabilitation

Prehabilitation is a multimodal strategy aimed at optimising patients’ physiological, physical, nutritional and psychological condition prior to surgery. This approach aims to enhance treatment tolerance, reducing the risk of complications and improve their overall functional status [70]. Some recent evidence exists supporting the implementation of prehabilitation in older cancer patients, demonstrating significant associations between higher cardiopulmonary exercise test (CPET) performance and higher peak oxygen uptake (VO_2_) values with improved postoperative outcomes, with fewer postoperative complications and shorter hospital stays [71], as well as improved functional and respiratory status following surgery [72]. A 2022 systematic review and meta-analysis by Guo et al. [18] investigated the effects of prehabilitation on postoperative outcomes in frail cancer patients undergoing elective surgery. Prehabilitation was associated with a 17% risk reduction in complications, a 38% risk reduction in severe complications and an average reduction in hospital stay of approximately 1.36 days compared to usual care, although no difference was seen in 30-day and 3-month mortality and readmission. Nevertheless, the results of this study highlight the benefits prehabilitation has on postoperative outcomes for older cancer patients.

#### Rehabilitation

After prehabilitation, rehabilitation is the next step in the continuum of optimisation for older cancer patients. Postoperative rehabilitation plays a pivotal role in improving recovery and quality of life following cancer surgery, especially if implemented early. In the PROLUCA (Postoperative Rehabilitation in Operation for LUng CAncer) clinical trial, a total of 235 patients with operable NSCLC were randomised to either early (14 days postoperatively) or late (14 weeks postoperatively) initiation of a 12-week structured rehabilitation program, comprising high-intensity interval and resistance training. The study observed that patients in the early rehabilitation group displayed continuous improvement in Health-Related Quality of Life (HRQoL) from baseline to 26 weeks. In contrast, patients in the late rehabilitation group experienced an initial non-significant deterioration in HRQoL during the first 14 weeks post-surgery. Although their HRQoL began to improve following the start of rehabilitation, it was unable to reach the levels observed in the early rehabilitation group at any point during the trial [20]. Consequently, these results indicate that early rehabilitation after cancer surgery could help to mitigate the impacts of surgery on quality of life and allow for faster recovery times.

## New Mindsets

### Patient preferences – function and Quality of Life

A shift in mindset in the way we approach treatment planning is important when implementing optimisation strategies. Traditionally, the focus of oncology has been on prolonging survival, but quality of life has become an increasingly important consideration, especially in the management of older patients.

A recent 2025 study by Morgan et al. [35] examined the treatment preferences of older women (aged ≥ 70 years) with early-stage breast cancer, particularly focusing on the balance between length of life and quality of life. The results showed that while both were highly valued, there was a small correlation between increasing age and relative quality of life preference. Additionally, younger individuals were found to be more likely to accept treatment-related toxicities in exchange for increased length of life. However, despite their tendency to avoid more aggressive treatments, length of life was still an important priority for women in the ≥ 80 age group, with preference scores comparable between the ≥ 80 and the 70–79-year age groups. By implementing strategies such as prehabilitation, which can mitigate postoperative outcomes, this could enable select older adults to pursue more aggressive treatment options, with no compromise in length or quality of life.

It is also important to note that a range of factors, including cancer type and demographics, can influence the prioritisation of treatment goals in older adults. For example, head and neck cancers are associated with significant decreases in functional status and IADL impairment 1 year after surgery [5]. A study by van Essen et al. [36] reported that over half (53.3%) of older adults with head and neck cancers identified maintaining independence as their primary treatment goal, while a smaller proportion prioritised extending life (34.1%), followed by pain reduction (8.2%) and the alleviation of other symptoms (4.1%). The study observed that patients living alone were more likely to prioritise reducing pain or other symptoms compared to patients living with family.

One approach to support shared decision-making for older adults includes the use of decision aids. Decision aids are tools which calculate personalised survival outcomes based on frailty, treatment modality, fitness and tumour stage. The Bridging the Age Gap study has demonstrated that for breast cancer, decision aids altered treatment choice towards PET instead of surgery for older adults, with no difference in survival between the two treatment options [73]. This provides evidence that decision aids can empower patients to be able to be more involved in the shared decision-making process, without compromising oncological outcomes. Additionally, validated questionnaires such as the Quality/Quantity – Early Cancer in the Elderly can help clinicians better understand patient preferences, with questions such as “I would always accept a hard to tolerate treatment, even if the chance of prolonging my life was small” and “I have lived my life and I don’t want to compromise the life I have with the treatment process” [35].

In order to assess surgeon attitudes towards the assessment and management of older cancer patients, the SIOG surgical task force conducted a survey on surgeons across America and Europe. When asked about the goal of treatment for older patients with cancer, quality of life and functional preservation were the primary objectives [74]. Furthermore, 71% of surgeons surveyed were prepared to prehabilitate and delay surgery for up to 4 weeks if proven to improve functional recovery, indicating a potential change in mindset, favouring quality of life over immediate intervention. Although the surgeons surveyed were those interested in geriatric oncology, this could indicate increased willingness to implement optimisation programs in older cancer patients.

### Multidisciplinary Team working – oncology and Geriatrics

Multidisciplinary team (MDT) working has become a standard component in modern cancer management, with specialised MDT meetings routinely convened across all cancer types to better individualise treatment plans. However, despite the widespread adoption of MDT collaboration, the integration of geriatrics into an oncology MDT remains relatively uncommon. Given the high prevalence of geriatric syndromes such as frailty and cognitive impairment in older patients with cancer, the involvement of geriatricians in an MDT could have the potential to better inform treatment decisions. This could help to optimise treatment selection and promote patient-centred outcomes, better addressing the needs of an ageing oncology population.

Recent evidence provides support for the benefits of MDT collaboration between oncology and geriatrics in improving outcomes for older cancer patients. A study in 2021 by Pang et al. [37] investigated the impacts of an MDT program on patients ≥ 65 with various cancers, who scored under ≤ 14 on the G8 geriatric screening tool, indicating frailty. A CGA was performed for all 544 patients, with interventions recommended by the MDT implemented accordingly. Among patients recommended for intervention, 92% received the proposed management. Notably, 31% of those who received interventions showed an improvement in their quality of life, and 29% of caretakers showed a reduced emotional/social/financial burden. Furthermore, patients enrolled in the MDT program had a shorter length of stay, averaging 10 days, compared to 15 days for non-enrolled patients. In another study by Mazzola et al. [38], patients with malignant tumours of the upper GI tract were assessed using the modified frailty index (mFI), after which identified frail patients underwent a multidisciplinary preoperative management plan, involving nutritional intervention, physical/respiratory enhancement and optimisation of existing treatment. The results demonstrated that the patients who received interventions had significantly lower 30-day and 3-month mortality and reduced overall and severe complication rates compared to a control group with similar frailty and surgical indications.

Despite this, multidisciplinary team working between surgical oncologists and geriatricians is limited. In the management of onco-geriatric surgical patients, the SIOG surgical task force survey found that 36.3% of surgeons surveyed never collaborate with geriatricians, and 33.5% involve geriatricians in under 25% of their patients. Additionally, only 48% consider a preoperative frailty assessment mandatory. Onco-geriatric assessments are also not commonly used, with only 6.4% of surgeons using CGA in daily practice, with non-onco-geriatric assessments used more frequently, in particular the American Society of Anaesthesiologists (ASA) score, performance status and nutritional status [74].

The following checklist provides a general structured framework to the assessment and management of older cancer patients. However, its applicability may vary across cancer types, and therefore recommendations should be adapted accordingly depending on cancer sites and treatment options.

1. Initial frailty screening for all patients ≥ 65.
  - G8, fTRST, CFS.
    i. G8 ≤ 14 [75] or fTRST ≥ 2 [17] or CFS ≥ 4 [76] – refer for CGA.
2. Physiological and comorbidity assessment
  - Assess physiological reserve using:
    i. CPET or VO₂ = predictor of postoperative complications [71, 77].
  - Comorbidity scoring:
    i. ECOG PS ≥ 2 = higher postoperative risk [17].
    ii. CACI ≥ 7 = impaired functional recovery and reduced QoL [17].
  - Offer prehabilitation to patients with evidence of reduced physiological reserve or significant comorbidity burden [18, 72].
3. Comprehensive Geriatric Assessment
  - Identify modifiable vulnerabilities.
    i. Tailor prehabilitation and rehabilitation to vulnerable domains [14–16].
4. Optimisation
  - Prehabilitation.
    i. Implement multimodal prehabilitation for ideally 4 weeks [74].
  - Perioperative considerations
    i. Delirium prevention [55, 56].
    ii. Early mobilisation [19, 54].
  - Postoperative rehabilitation
    i. Start early structured rehabilitation ideally within 2 weeks [20].
5. Explore patient preferences
  - Utilise validated decision aids to support understanding of treatment options and preferred outcomes [73].
6. Multidisciplinary decision making.
  - Integrate CGA findings, physiological reserve, tumour biology, operability and feasibility of adjuvant therapies, patient goals and risk tolerance with input from geriatrics [17, 37, 38].
7. Treatment selection.
  - Curative intent.
    i. Offer standard surgical treatment when:
      1. Tumour is potentially curable.
      2. Adequate physiological reserve.
      3. Oncologic benefit outweighs perioperative risk [77–79].
    ii. Prefer minimally invasive surgery where appropriate [77–79].
  - Locally advanced disease.
    i. Assess suitability for multimodal therapy (e.g. neoadjuvant chemotherapy) based on:
      1. Tumour biology.
      2. Frailty.
      3. Comorbidities.
      4. Ability to complete systemic therapy [78–80].
    ii. Avoid neoadjuvant therapy when:
      1. Marked frailty.
      2. High risk of not completing treatment or reaching surgery [31].
    iii. Consider upfront surgery followed by adjuvant therapy in borderline-operable patients [79].
  - Non-operative alternatives.
    i. Offer non-operative approaches according to:
      1. Eligibility for surgery.
      2. Tumour-specific evidence.
      3. High postoperative risk.
      4. Patient preferences [78, 79].

## Future Directions

Older cancer patients remain underrepresented in clinical trials, limiting the generalisability of existing evidence and contributing to uncertainty around best treatment practices within this population. Future clinical trials focusing on the older cancer population can help to better inform treatment selection.

Further evidence is needed to determine which components of prehabilitation and rehabilitation, such as exercise training, nutritional optimisation, and psychological support contribute most significantly to improved outcomes, and how these elements can be best tailored to provide optimal outcomes in older cancer patients.

Additionally, the routine use of geriatric assessment and MDT collaboration between oncologists and geriatricians remains relatively low despite growing evidence to support its benefits. As the evidence base continues to expand, future research and clinical guidelines should aim to standardise the incorporation of geriatric principles into surgical oncology to deliver more holistic, patient-centred care for this growing and vulnerable patient population. Further research on the cost-effectiveness of CGA is needed to assess whether the benefits of CGA outweigh the costs associated.

## Limitations

This review has several limitations. It is a non-systematic narrative review, and therefore studies were selected based on author judgement rather than with an inclusion and exclusion criteria, which may introduce selection bias. The search strategy also prioritised English-language publications from the past five years, potentially excluding relevant older or non-English studies.

Secondly, the evidence base itself is highly heterogeneous, with multiple cancer types, treatment modalities, and outcome measures. This variability limits direct comparability across studies and limits the ability to recommend definitive conclusions regarding best surgical practices for older adults. Furthermore, many referenced randomised controlled trials were not specifically designed for older or frail populations, who are frequently underrepresented or excluded from clinical trials. As a result, this limits the extrapolation of trial findings to real-world older cancer patients, and therefore should be carefully considered when interpreting the evidence presented.

Finally, as a narrative synthesis, this review provides a thematic overview, and should therefore be interpreted as broad trends within the current literature.

## Conclusions

While surgery remains a cornerstone of cancer treatment, alternative approaches such as non-operative and minimally invasive techniques have demonstrated comparable oncological outcomes in selected populations, with the added benefit of reduced perioperative morbidity and better preservation of function. These advancements expand the available treatment options and are important alternatives to consider, considering patient preferences and impacts on quality of life. Careful patient selection is essential to identify those who are suitable candidates for these alternative approaches, ensuring optimal outcomes while minimising risk.

The integration of geriatric assessments, frailty screening tools, and prehabilitation protocols into routine surgical planning has shown promise in reducing postoperative complications, enhancing recovery, and improving overall quality of life. Incorporating geriatric expertise within surgical MDTs has also been associated with meaningful modifications to treatment plans. Prehabilitation and rehabilitation strategies offer further opportunities to mitigate postoperative complications and promote functional recovery, reflecting a shift towards not only extending life but improving quality of life.

## Data Availability

No datasets were generated or analysed during the current study.

## Key References

- Montroni I, Ugolini G, Saur NM, Rostoft S, Spinelli A, Van Leeuwen BL, et al. Predicting Functional Recovery and Quality of Life in Older Patients Undergoing Colorectal Cancer Surgery: Real-World Data From the International GOSAFE Study. J Clin Oncol. 2023;41(34):5247-62. doi: 10.1200/JCO.22.02195.
  - ○ Identified frailty as a key predictor of quality of life and functional recovery following colorectal cancer surgery.

- Buchberger DS, Videtic GMM. Stereotactic Body Radiotherapy for the Management of Early-Stage Non-Small-Cell Lung Cancer: A Clinical Overview. JCO Oncol Pract. 2023;19(5):239 − 49. doi: 10.1200/OP.22.00475.
  - ○ An overview of the use of SBRT in NSCLC, detailing evidence supporting its safety and efficacy in non-operable NSCLC.

- Goede V. Frailty and Cancer: Current Perspectives on Assessment and Monitoring. Clin Interv Aging. 2023;18:505-21. doi: 10.2147/CIA.S365494.
  - ○ This narrative review presents a summary of frailty assessment methods currently in practice and evaluates their effects on clinical outcomes.

- Horiuchi K, Kuno T, Takagi H, Egorova NN, Afezolli D. Predictive value of the G8 screening tool for postoperative complications in older adults undergoing cancer surgery: A systematic review and meta-analysis. J Geriatr Oncol. 2024;15(3):101656. doi:10.1016/j.jgo.2023.101656.
  - ○ This systematic review demonstrates that the G8 is an effective predictor of postoperative complications in older cancer patients.

- Morgan JL, Shrestha A, Martin C, Walters S, Bradburn M, Reed M, et al. Preferences for quality of life versus length of life in older women deciding about treatment for early breast cancer: A cross-sectional sub-analysis of the Bridging the Age Gap study. J Geriatr Oncol. 2025;16(4):102226. doi: 10.1016/j.jgo.2025.102226.
  - ○ This study identifies that both life extension and quality of life are important treatment goals for older women with early breast cancer, with relative QoL preference increasing with advancing age. Older patients were also less likely to accept treatment-related toxicities in exchange for increased length of life.
